# Economic Analysis of Alternative Strategies for Detection of *ALK* Rearrangements in Non Small Cell Lung Cancer

**DOI:** 10.3390/diagnostics6010004

**Published:** 2016-01-06

**Authors:** Shivang Doshi, David Ray, Karen Stein, Jie Zhang, Prasad Koduru, Franz Fogt, Axel Wellman, Ricky Wat, Charles Mathews

**Affiliations:** 1 Boston Healthcare Associates, 75 Federal Street, Boston, MA 02110, USA; ricky.wat@jhu.edu (R.W.); cmathews@bostonhealthcare.com (C.M.); 2 Ernest Mario School of Pharmacy, Rutgers University, 160 Frelinghuysen Rd, Piscataway Township, NJ 08854, USA; david.ray@novartis.com; 3 Novartis Pharmaceuticals Corporation, One Health Plaza, East Hanover, NJ 07936, USA; karen.stein@novartis.com (K.S.); jie.zhang@novartis.com (J.Z.); 4 UT Southwestern Medical Center, 2330 Inwood Road, Dallas, TX 75235, USA; Prasad.Koduru@UTSouthwestern.edu; 5 Penn Presbyterian Medical Center, Department of Pathology, Philadelphia, PA 19104, USA; Franz.Fogt@uphs.upenn.edu; 6 The Pathological Institute Celle, Wittinger Str. 14, 29223 Celle, Germany; axel.wellmann@pathologen.net; 7 Johns Hopkins University, 3400 N. Charles Street, Baltimore, MD 21218, USA

**Keywords:** Anaplastic lymphoma kinase (*ALK*) gene rearrangement, immunohistochemistry (IHC), fluorescent *in situ* hybridization (FISH), non-small cell lung cancer (NSCLC), cost-impact model

## Abstract

Identification of alterations in *ALK* gene and development of *ALK*-directed therapies have increased the need for accurate and efficient detection methodologies. To date, research has focused on the concordance between the two most commonly used technologies, fluorescent *in situ* hybridization (FISH) and immunohistochemistry (IHC). However, inter-test concordance reflects only one, albeit important, aspect of the diagnostic process; laboratories, hospitals, and payors must understand the cost and workflow of *ALK* rearrangement detection strategies. Through literature review combined with interviews of pathologists and laboratory directors in the U.S. and Europe, a cost-impact model was developed that compared four alternative testing strategies—IHC only, FISH only, IHC pre-screen followed by FISH confirmation, and parallel testing by both IHC and FISH. Interviews were focused on costs of reagents, consumables, equipment, and personnel. The resulting model showed that testing by IHC alone cost less ($90.07 in the U.S., $68.69 in Europe) than either independent or parallel testing by both FISH and IHC ($441.85 in the U.S. and $279.46 in Europe). The strategies differed in cost of execution, turnaround time, reimbursement, and number of positive results detected, suggesting that laboratories must weigh the costs and the clinical benefit of available *ALK* testing strategies.

## 1. Introduction

Lung cancer is the leading cause of cancer-related death worldwide [1]. Over 1.7 million individuals are living with lung cancer globally. In the United States, over 220,000 new cases of lung cancer are diagnosed, and over 150,000 people die annually, accounting for nearly 30% of all cancer deaths [2]. In Europe, there are over 400,000 new lung cancer cases with approximately 370,000 deaths each year. Non small cell lung cancer (NSCLC) accounts for 85% of all lung cancers globally and has a poor prognosis because most patients have advanced disease at the time of diagnosis.

Ongoing development of molecularly targeted therapies, such as afatinib (Gilotrif, Boehringer Ingelheim, Ingelheim, Germany), gefitinib (Iressa, AstraZeneca, London, UK) and erlotinib (Tarceva, Roche, Basel, Switzerland), has given rise to practice-changing developments in patient care. Predictive biomarkers of therapeutic-sensitive genetic mutations are undergoing clinical validation studies, and results have been promising [3]. As a result, clinically actionable biomarkers are increasingly adopted in routine clinical care. The current paradigm is to select, prescribe, and deliver care to patients most likely to benefit from targeted therapy [4].

Genetic aberrations in anaplastic lymphoma kinase (*ALK*) have recently generated interest in developing targeted therapies due to their role in tumor growth. The two primary *ALK* alterations of clinical importance are point mutations and rearrangements. *ALK* rearrangement occurs in 2%–11% of all NSCLC cases [5]. Crizotinib (Xalkori, Pfizer Oncology, New York, NY, USA), the first approved *ALK* inhibitor, had an overall response rate (ORR) of 60% and a superior median progression-free survival (mPFS) of 7.7 months compared to standard chemotherapy treatment with mPFS of 3.0 months [6]. Ceritinib (Zykadia, Novartis, Basel, Switzerland), a second-generation *ALK* tyrosine kinase inhibitor, was approved by the U.S. Food and Drug Administration (FDA) and European Medicines Agency (EMA) for *ALK*-positive patients who progressed on or were intolerant to crizotinib treatment. The pivotal study demonstrated an ORR of 61.8% and a mPFS of 9.0 months in *ALK*-positive patients regardless of treatment with an *ALK* inhibitor. Ceritinib achieved an ORR of 56.4% in crizotinib-treated patients and 72.3% in crizotinib-naïve patients. The mPFS was 6.9 months in crizotinib-treated patients and 18.4 months in crizotinib-naïve patients [7,8].

Currently, national guidelines and specialty societies support testing all lung adenocarcinomas for *ALK* aberrations, irrespective of other variables such as race, sex and smoking history [9]. IASLC recommends *ALK* testing in patients with Stage IV lung cancer and encourages it in patients with Stage I, II or III lung cancer [10]. Similarly, National Comprehensive Cancer Network (NCCN) and European Society for Medical Oncology (ESMO) recommend all patients with lung adenocarcinoma to be tested for *ALK* rearrangements, among other genetic alterations [11,12].

Characterization of the role of *ALK* rearrangements and emergence of *ALK*-directed therapies necessitates assays to detect *ALK*-gene rearrangements. Currently available methods differ with respect to equipment and personnel requirements, workflows and turn-around times, analytical parameters (sensitivity and specificity), and cost of execution.

Break-apart fluorescent *in situ* hybridization (FISH), the standard method to detect *ALK* rearrangements, binds a probe to the 2p23.2 region of *ALK* gene to detect rearrangement. There are a few *ALK* FISH probes and automated slide staining platforms available in the market. Abbott’s Vysis LSI *ALK* Break Apart FISH probe kit (Abbott Molecular, Des Plaines, IL, USA) is the most widely used *ALK* probe, especially in the U.S., where the kit is FDA-approved as a companion diagnostic for an *ALK* inhibitor [13]. Cytocell and Zytovision have also developed *ALK* breakapart probe sets.

FISH workflow can be manual or automated. For example, Abbott’s VP2000 (Abbott Molecular) and Leica Biosystems’ BOND-III (Leica Biosystems, Wetzlar, Germany) processors automate deparaffinization, pretreatment, staining and slide washing and can run 30–50 slides in a single run [14]. Even fluorescent visualization of the probe can be automated by using a scanning station. BioView’s Duet-3 (BioView, Billerica, MA, USA) is an example of an automated scanning station.

Immunohistochemistry (IHC) offers an alternative method to detect *ALK* abnormalities. Unlike FISH that detects *ALK* gene rearrangements directly, IHC detects the aberrant protein resulting from the different rearrangements. Laboratories use a variety of anti-*ALK* primary antibodies and automated platforms. In June 2015, the FDA approved a rabbit monoclonal antibody D5F3 (Ventana/Cell Signaling Technology, Danvers, MA, USA) that may be used to determine treatment eligibility for Xalkori. Other available antibodies include two mouse monoclonal antibodies clones, ALK1 (Dako, Agilent Technologies, Santa Clara, CA, USA) and 5A4 (Novocastra, Leica Biosystems, Wetzlar, Germany) and a rabbit polyclonal antibody (Invitrogen, Life Technologies, Carlsbad, CA, USA). IHC can be executed manually or by an automated slide staining system, such as Ventana’s BenchMark XT (Roche, Basel, Switzerland) and Leica Biosystem’s BOND-III (Leica Biosystems, Wetzlar, Germany). Automated platforms allow laboratories to increase batch size, simultaneously stain slides with multiple antibodies, and significantly reduce labor times and costs.

RT-PCR and next-generation sequencing (NGS) are also deployed by molecular laboratories, individually or in combination with FISH or IHC. RT-PCR detects *ALK* rearrangements in NSCLC patients but requires high-quality RNA, which may be difficult to obtain from small formalin-fixed paraffin-embedded (FFPE) biopsies in clinical practice [4] Moreover, RT-PCR requires primer-sets for each of the many different variants of *ALK* translocations and, therefore, continuous updating of the primers when new variants are discovered [15,16]. Therefore, RT-PCR is not recommended for screening of patients for treatment with an *ALK* inhibitor [4]. NGS on the other hand has the advantage of being able to detect different variants of *ALK* translocations from a single sample. It can also simultaneously detect aberrations in other genes, making it an attractive methodology for targeted therapy selection in the future. However, laboratories running large NGS panels still rely on IHC or FISH to accurately detect *ALK* rearrangements.

With alternative assays available for detection of *ALK* rearrangements, health systems, laboratories, healthcare professionals and payers need to compare the workflow, accuracy, costs, and reimbursement of the various testing approaches to identify the most cost-effective approach for their setting.

## 2. Methods Section

The current study reviewed the literature appraising *ALK* testing in NSCLC and interviewed laboratories in the U.S. and Europe that routinely perform *ALK* testing on NSCLC samples to create a payer-directed cost-impact model. Abstracts of research studies and review articles appraising the analytical validity, economic impact and clinical benefit of *ALK* testing were reviewed and selected for full-text review to source key model inputs on prevalence of lung cancer, rates of *ALK* positive results by IHC or FISH, assay failure rates, and concordance between IHC and FISH. A total of 40 research articles were reviewed. Additionally, IHC and FISH reimbursement rates in the U.S. were obtained from the Medicare physician fee schedule [17], in Europe, some markets have a budget-based payment system (UK, Spain) while some have a fee schedule-based payment system (France, Germany). We obtained the average payment on a per test basis from the laboratories we interviewed, and calculated an average reimbursement rate for *ALK* testing in Europe.

Focused telephone interviews using a structured questionnaire captured information on *ALK* testing techniques and materials, test volumes, batch size, test configuration (platforms, kits), average turn-around times, assay workflow, and use of resources/supplies (reagents, consumables, equipment, and personnel). The cost of reagents was calculated by multiplying the per unit cost with the total quantity used per batch. Similarly, the cost of labor was calculated by multiplying the per hour rate with the hands-on time per batch. Equipment cost was derived by assigning a useful life to each piece of equipment and amortizing the price of the equipment to a per minute rate and multiplying it with the use time per batch (Equations 1–3). The cost inputs were then incorporated into the model. The endpoints of the model were the number of patients detected with an *ALK* rearrangement by each testing strategy, total cost per identified sample, and the expected reimbursement to the laboratory.

Cost of reagents/consumables = (quantity used per batch × unit cost) ÷ batch size
(1)

Cost of equipment = (equipment price ÷ useful life) × use time per batch ÷ batch size
(2)

Cost of labor = hands-on time per batch in hours × hourly pay rate ÷ batch size
(3)

## 3. Results

A total of 10 laboratories qualified and agreed to participate in the study (three in the U.S., three in Germany, two in Spain, one in France and one in UK). Annual lung cancer-specific *ALK* testing volume ranged from 150 to 1500 among laboratories in the U.S., and from 200 to 3600 among laboratories in Europe.

**Figure 1:**
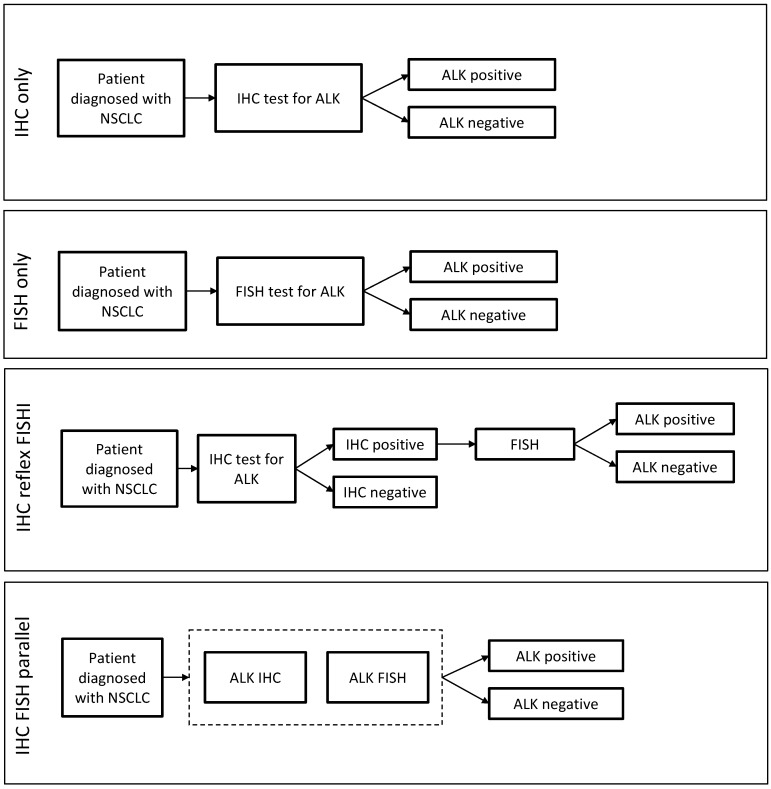
Alternative testing strategies employed by laboratories included in the research for *ALK* rearrangement detection in non small cell lung cancer (NSCLC) patients. The strategies comprise of fluorescent *in situ* hybridization (FISH) and immunohistochemistry (IHC) individually or in combination. In the “IHC reflex FISH” testing strategy, only patients that test positive via IHC and FISH are considered positive for *ALK* rearrangement and would be eligible for TKI treatment in the model. In “IHC FISH parallel” strategy, the dash line box indicates use of both IHC and FISH in parallel, and in no particular order, to determine ALK status.

Literature review and primary research with laboratories identified four most widely used approaches to *ALK* testing involving IHC and FISH, either individually or in combination, as represented in Figure 1. Using the key parameters listed in Table 1, we determined the number of *ALK* rearrangement detections, average cost and reimbursement in the U.S. and Europe. Additionally, the time to result for IHC and FISH was obtained via interviews with laboratorians. These are summarized in Table 2. Among labs interviewed, nearly all laboratories currently rely primarily on FISH or IHC to detect *ALK* rearrangements.

**Table 1:** Key model parameters for cost-impact model.

Variable	Value	Lower Limit	Upper Limit	Source
Cost of IHC per sample (USA)	$89	$54	$124	Lab. survey
Cost of IHC per sample (Europe)	$67.88	$33	$112.73	Lab. survey
Cost of FISH per sample (USA)	$330	$300	$360	Lab. survey
Cost of FISH per sample (Europe)	$197.72	$169.09	$244.20	Lab. survey
IHC reimbursement (USA)	$90.46	-	-	Medicare [17]
FISH reimbursement (USAA)	$216.35	$214.52	$217.38	Medicare [17]
*ALK* testing reimbursement (Europe)	$155.50	$132	$186	-
Probability of *ALK*+ result by IHC	4.0%	3.4%	10.1%	Paik [18], Cabillic [19], Ali [20], Sullivan [21]
Probability of *ALK*+ result by FISH	4.2%	3.8%	6.4%	Paik [18], Cabillic [19], Ali [20], Sullivan [21]
Probability of *ALK*+ result by either IHC or FISH	4.9%	3.8%	10.1%	Paik [18], Cabillic [19], Ali [20], Sullivan [21]
Probability of *ALK*+ result by IHC but *ALK*− by FISH	0.7%	0.0%	3.7%	Paik [18], Cabillic [19], Ali [20], Sullivan [21]
Failure rate of *ALK* IHC	1.2%	-	-	Zhou [22]
Failure rate of *ALK* FISH	6.6%	-	-	Zhou [22]

IHC, immunohistochemistry; FISH, fluorescent *in situ* hybridization.

**Table 2:** Results summary from the cost-impact model (all costs in 2015 United States Dollars).

Testing Strategy	Time to Result (Working Days)	*ALK* Positivity Rate	US		Europe	
			Average Cost (USD)	Average Reimbursement * (USD)	Average Cost (USD)	Average Reimbursement ** (USD)
IHC only	1 to 2 days	4.0%	$90.07	$91.55	$68.69	$157.37
FISH only	2 to 5 days	4.2%	$351.78	$230.63	$210.77	$165.76
IHC reflex FISH	1 to 2 days if IHC−; 3 to 7 days if IHC+	3.3%	$104.12	$100.76	$77.11	$157.37
IHC FISH parallel	2 to 5 days	4.9%	$441.85	$322.17	$279.46	$157.37

* The U.S. reimbursement rate is based on the Medicare 2015 Physician Fee Schedule; CPT codes 88,342 for IHC, 88,367 and 88,368 for FISH. ** In contrast to the methodology- and fee schedule-based system in the U.S., reimbursement in Europe could be either fee schedule-based or budget-based, and could be specific to the biomarker (ALK) or to the methodology.

### 3.1. Comparison of Workflow

**Figure 2:**
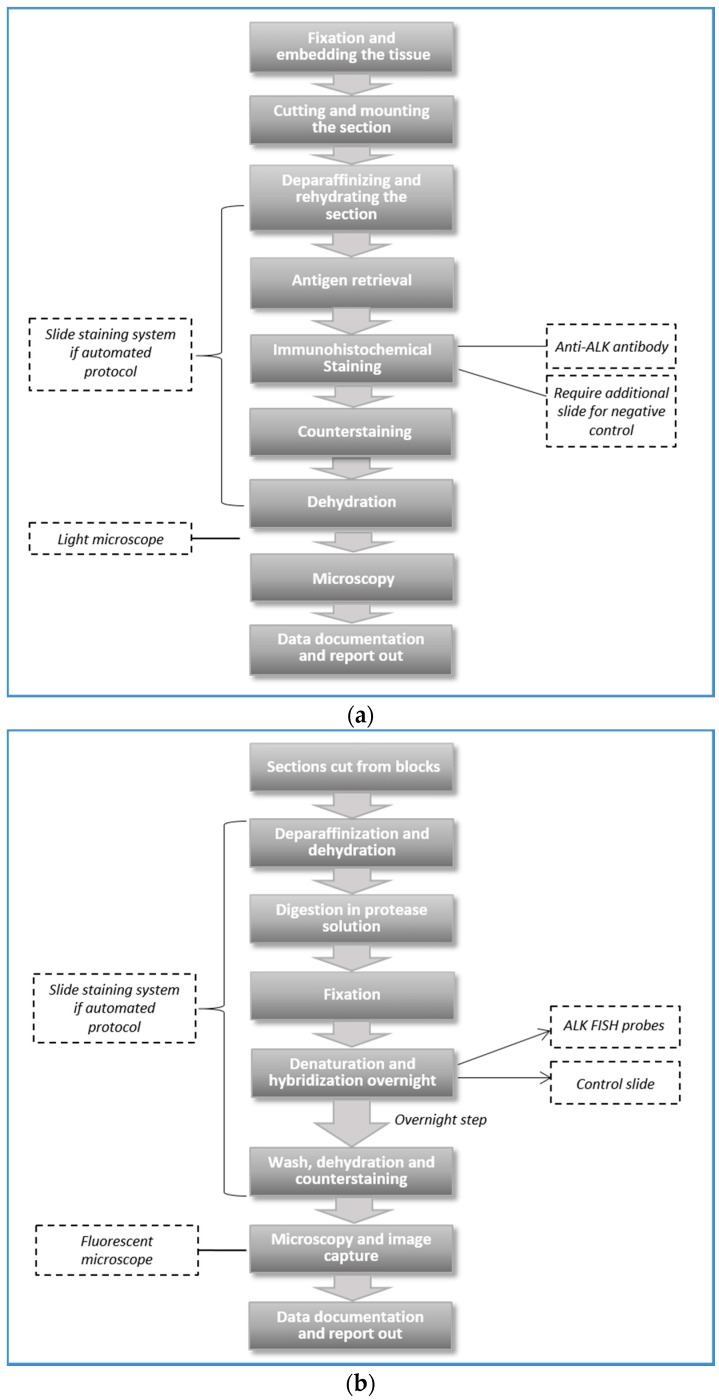
Key steps in the protocol of (**a**) IHC and (**b**) FISH methodologies.

IHC and FISH protocols (Figure 2a,b) overlap in some aspects of workflow, such as slide sectioning, processing, incubation (with anti-*ALK* antibody or FISH probe), and visualization under light (IHC) or fluorescent (FISH) microscope [23]. However, many institutions perform IHC and FISH at different sites either within a single department, or between different entities (*i.e.*, cytogenetic laboratory), or at outside institutions. The turnaround time ranges from one to two working days for IHC to two to five working days for FISH. The “IHC only” strategy required the shortest turnaround time of 1 to 2 working days. “FISH only” and “IHC FISH parallel” strategies required two to five working days. The “IHC reflex FISH” strategy needed seven working days for FISH confirmation of a positive IHC result and up to two working days for a negative IHC result (Table 2).

In practice, turnaround time for *ALK* analysis varies widely and can require up to three weeks. Other activities contribute to turnaround time, irrespective of the technique used: test requisition, sample collection and transportation, pathology, confirmation of adequate tissue, and reporting results. Time for non-test specific activities varies by institutional organization.

Thus, the comparison of turnaround times above between the *ALK* testing strategies is relative rather than the absolute turnaround times of the approaches.

### 3.2. Comparison of ALK Testing Costs

Through primary research with laboratories, we obtained information on cost of reagents and consumables, equipment and labor that are involved in *ALK* IHC and FISH assays. Reagents include anti-*ALK* antibodies (for IHC) and probes (for FISH); equipment includes light or fluorescent microscopes, automated processors. Labor includes technicians and pathologists.

The average cost of *ALK* IHC was $72.58 per sample (range: $33 to $124) depending on antibody and platform, batch size, and batch efficiency. The average cost was $89 among the U.S. labs and $67.88 among European labs. The lowest cost, $33, came from a laboratory that ran IHC slides from multiple tumor types and for various biomarkers in a single batch of 30 samples using Ventana’s Benchmark XT. On the other hand, the cost was $124 for a laboratory that ran a single *ALK* IHC slide, but the cost decreased to $54 when batch size increased from one to five.

For FISH, the average per sample cost was $227.12 (range: $169.09 to $360). The average cost was $330 among the U.S. and $197.72 among European labs. As with IHC, the cost of FISH depended on the choice of probes and platform, batch size, and batch efficiency. In the U.S., Abbott’s (Des Plaines, IL, USA) *ALK* probe kit cost more than the Cytocell (Cambridge, UK) probe set ($360 *vs.* $300 per sample). In Europe, Abbott’s probe cost more than Zytovision (Bremerhaven, Germany) probes ($205 *vs.* $189 per sample).

Antibodies for IHC, FISH probes, and labor drive *ALK* testing costs. Typically, laboratories negotiate discounted rates on the reagents and equipment and rarely pay the list price. Different discounts and contracting partly explain the variability observed across laboratories. Both IHC and FISH assays require capital expenditure (e.g., light and fluorescent microscopes, automated slide staining systems, computers), but the equipment is not exclusively used for *ALK* testing. Hence, the amortized cost of equipment per sample may be less than 1% of the total cost per sample and was not considered a significant cost driver.

The “IHC only” strategy was the least expensive approach (average cost per sample of $90.07 in the U.S. and $68.69 in Europe, Table 2). This is not unexpected as average IHC cost per sample is over 3 times lower than the average FISH cost per sample. The “IHC FISH parallel” strategy (IHC and FISH on all patients) is the most expensive (average cost of $441.85 in the U.S. and $279.46 in Europe). Irrespective of location, the “IHC reflex FISH” strategy in which FISH is performed only on patients who test positive by IHC is only slightly more expensive than the “IHC only” strategy.

### 3.3. Reimbursement for ALK Testing

In the U.S., reimbursement to laboratories is fee schedule-based. Both FISH and IHC are on the Medicare fee schedule: $216 and $90, respectively [24]. FISH reimbursement rates recently were cut by almost 60% compared to the 2014 rates. Commercial health plans typically pay more than Medicare and are separately and confidentially negotiated. Thus, average reimbursement rates listed for each strategy are based on Medicare fee schedule only.

In Europe, payment for *ALK* testing comes from multiple sources, including insurance companies, drug manufacturers, and the global hospital budget. Moreover, payment is often independent of the test methodology and specific to the biomarker. For example, drug manufacturers pay the laboratory a fixed fee (€120) for *ALK* testing in Spain, irrespective of test methodology. Similarly, the French Institut National Du Cancer (INCa) pays a fixed amount (€135) for *ALK* testing. To contain costs and maintain net margins, laboratories in Spain and UK both have shifted to an “IHC reflex FISH” strategy from “FISH only” or “IHC FISH parallel” strategies.

### 3.4. Detection of ALK Positives by Strategy

The “IHC FISH parallel” strategy results in the highest detection rate of *ALK* positivity. The “IHC reflex FISH” strategy limits patient identification by excluding patients tested positive by IHC but negative by FISH (Table 2). The “IHC only” strategy and “FISH only” identifies similar numbers of *ALK* positive patients but with some false negative and false positive results. Health systems must weigh the overall clinical benefit of maximizing use of *ALK* targeted therapy with the risks of treating false positive patients.

## 4. Discussion

Combining the review of the literature with field research in the U.S. and in Europe allowed development of a cost-impact model to compare alternative *ALK* testing strategies in terms of the cost of execution, reimbursement rates, workflow, turnaround times, and the number of *ALK* rearrangements identified. According to the model, testing approaches which included either IHC or an IHC pre-screen followed by confirmation with reflex FISH cost least but with significant variation in turnaround time. Pre-screening with IHC also is limited by the sensitivity of IHC and the specificity of FISH in identifying cases eligible for targeted treatment. Meanwhile, parallel testing by both IHC and FISH results in higher costs to the laboratory and the possibility of discordant results. The key cost drivers are the antibodies (IHC) and probes (FISH), and personnel costs for laboratory staff, including technicians, histotechnologists, and pathologists. We recognize that there are differences in the choice of antibodies, probes, platforms, level of automation, and involvement of laboratory staff, all of which would have an impact on the cost of different *ALK* testing strategies. The range of cost of *ALK* IHC and FISH tests, in the U.S. and in Europe, are listed in Table 1. Hence, the total cost of the different *ALK* strategies will change depending on factors listed above, though it is unlikely to have an impact on the directionality of our findings from the model.

Laboratories also aim to optimize the testing workflow. The “IHC only” strategy yields the quickest turnaround time (one to two working days), while a sequential approach of pre-screening with IHC followed by FISH in IHC positive results yields a turnaround time of up to seven working days. Molecular testing guidelines released by the College of American Pathologist, the International Association for the Study of Lung Cancer, and the Association of Molecular Pathology recommend that the test results be available within two weeks (10 working days) from the time the lab receives the specimen [10]. However, the overall turnaround time for *ALK* testing may require up to three weeks, if other components of the workflow such as test requisition, sample collection and transport are included.

Numerous studies have examined the concordance of IHC and FISH for *ALK* rearrangements. Cabillic *et al.* [19], found only 53% of cases classified as *ALK* positive by both IHC and FISH assays. In the study, of 114 IHC positive cases, 36 were either negative or inconclusive with FISH, probably due to presence of variants not detectable by FISH probes. Blackhall *et al.* [25], found similar results among 240 non-squamous cell carcinomas of the lung. Rodig *et al.* [26], concluded that approximately 20% of FISH positive cases will not produce a positive result by IHC, which are missed if FISH testing was limited to the confirmation of positive IHC tests only. It should be noted that greater IHC testing sensitivity is possible with the advent of more sensitive antibodies such as the rabbit monoclonal antibody D5F3 [27]. The overall response rates of currently available treatments suggests that approximately 40% of identified patients using FISH alone will not respond, perhaps due to the fact that not all *ALK* rearrangements result in expressed *ALK* fusion proteins. This implies that a dual strategy, whether parallel or reflex, may have the potential to alter these rates. However, the reflex IHC with FISH confirmation may screen out eligible patients. Although rare, previous literature has reported FISH insensitivity to certain *ALK* translocations identified through IHC testing [28,29]. Thus, the slowest and most expensive approach may offer the best probability of detecting cases.

Far fewer studies have reported the cost of *ALK* testing to the laboratory. Parker *et al.* developed a micro-cost model to estimate the cost of the *ALK* FISH test ($278.01), similar to our finding via the laboratory survey [30]. Lee *et al*.’s [31] analysis relied on the French reimbursement tariff for FISH ($175 per test) as the cost input, rather than the actual cost of execution [31]. Atherly *et al.* [32] used billed charges ($1400 per test) and may have overestimated the cost of *ALK* testing.

While this study attempted to measure the actual cost of *ALK* testing, several limitations should be noted. First, the costs of IHC and FISH were based on telephone interviews with laboratory directors or pathologists, not on assay protocols from the laboratories. Second, the model did not directly assess the clinical validity of the testing strategies. Based on rates of IHC and FISH positivity in literature, we calculated the number of *ALK* rearrangement detections in a sample of 1000 patients. However, the model did not predict the false positive and false negative rates, nor the consequent impact on clinical benefit of *ALK* targeted therapies. Finally, the model did not consider emerging or alternative *ALK* testing methods such as RT-PCR and NGS.

## 5. Conclusions

In conclusion, the model provides real-world insights into the cost, reimbursement, workflow, and potential number of patients on *ALK* targeted therapy associated with alternative *ALK* testing strategies, comprising of FISH and IHC. The analysis includes differences in geographical contexts, reagents and platforms, and other assay specifics. We intend laboratories and health systems to utilize this study to assess different testing options available to them, and weigh their costs and clinical benefits, with the goal of enhancing access to available therapy options to patients.
